# Gut Microbiota and Immunotherapy

**DOI:** 10.3389/fmicb.2022.945887

**Published:** 2022-07-01

**Authors:** Xiaoqing Xu, Jieer Ying

**Affiliations:** ^1^Department of Medical Oncology, The Second Clinical Medical College of Zhejiang Chinese Medical University, Hangzhou, China; ^2^Department of Hepato-Pancreato-Biliary and Gastric Medical Oncology, Cancer Hospital of the University of Chinese Academy of Sciences (Zhejiang Cancer Hospital), Institute of Basic Medicine and Cancer (IBMC), Chinese Academy of Sciences, Hangzhou, China

**Keywords:** immunotherapy, the gut microbiota, immunotherapy response, interaction, microbiome manipulation

## Abstract

The gut microbiota is the largest microbiota in the body, which is closely related to the immune state of the body. A number of studies have shown that gut microbiota and its metabolites are involved in host immune regulation. Immune checkpoint inhibitors have become an important drug for the treatment of many malignant tumors, which can significantly improve the prognosis of tumor patients. However, a considerable number of patients cannot benefit from immune checkpoint inhibitors. At present, the known treatment methods of microbiota manipulation mainly include fecal microbiota transplantation, dietary regulation, prebiotics and so on. Therefore, this paper will discuss the possibility of improving the anti-tumor efficacy of immunotherapy from the perspectives of the gut microbiota and immunotherapy.

## Introduction

A large number of microorganisms exist in the human intestinal tract, including bacteria, viruses and fungi, which together constitute the gut microbiota (Human Microbiome Project C, 2012; Sarin et al., 2019). The gut microbiota interacts with the body, participating in the digestion and metabolism, affecting the body’s immunity and the formation of diseases. Studies have found that a highly diverse gut microbiota creates a symbiotic relationship with the body’s immune system, promoting homeostasis, and the disruption of homeostasis can lead to chronic inflammation, autoimmune diseases, and even cancer (Ticinesi et al., 2019; Lavelle and Sokol, 2020; Zhong et al., 2020; Isacco et al., 2021). In recent years, the research of immunotherapy mainly focuses on the application of immune checkpoint inhibitors (ICIs), including antibodies against cytotoxic T lymphocyte antigen-4 (CTLA-4), programmed cell death protein 1 (PD-1) and programmed death ligand 1(PD-L1). At present, ICIs has gradually become an indispensable method in the treatment of hematological tumors and a variety of solid malignancies, and has achieved remarkable efficacy (Ribas and Wolchok, 2018). Although immunotherapy has many advantages, the effective rate of immune checkpoint inhibitors is not high, and different patients also have great differences in response to treatment Human Microbiome Project C, 2012. This paper discusses the relationship between the gut microbiota and immunotherapy.

## Gut Microbiota

For the recent years, the gut microbiota has become one of the hot topics in medicine, microbiology, ecology, and genetics. The gut microbiota is a very complex ecosystem, with more than 2,000 species, which mainly include the bacteria, viruses, and fungi. On the basis of the function and nature of gut microbiota, it can be roughly divided into three types: the beneficial bacteria, the unstable condition pathogenic bacteria, and the pathogenic bacteria (Blaser and Falkow, 2009; Montalban-Arques and Scharl, 2019). Beneficial bacteria can reduce the production of cholesterol, synthesize a variety of vitamins, promote intestinal peristalsis, improve immunity, etc., with *Bacteroidetes, Clostridium, Bifidobacterium, Lactobacillus* as the representative; Pathogenic bacteria in unstable conditions are beneficial to health under normal circumstances. However, under certain conditions such as low immunity and weak constitution, they may proliferate out of control or transfer from the intestine to other parts of the body, leading to intestinal damage, which is represented by *Escherichia coli* and *Enterococcus*. Pathogenic bacteria refers to the bacteria that are not inherent in the intestinal tract (Round and Mazmanian, 2009; Kamada et al., 2013; Baumler and Sperandio, 2016). Once getting out of control, it will affect the immunity of the body and the formation of diseases, such as *Salmonella* and *pathogenic E. coli* (Panda et al., 2014). Studies have found that the highly diverse gut microbiota establishes a symbiotic relationship with the body’s immune system. Disruption of this relationship may lead to tumor initiation and development (Casey et al., 2014; Poutahidis and Erdman, 2016; Denton et al., 2018; Mantovani et al., 2019). Deborah Nejman et al. analyzed seven cancer types (breast, lung, ovarian, pancreatic, melanoma, bone, and brain tumors) and found that different types and subtypes of tumors have different microbiome compositions (Nejman et al., 2020). The relative contents of *Bacillus, Enterobacteriaceae,* and *Staphylococcus* were higher in the breast tissue of the patients who suffer from the breast cancer (Urbaniak et al., 2016). Patients who suffer from the estrogen receptor-positive breast cancer, had higher concentrations of arsenic detoxifying microorganisms than those with estrogen receptor-negative breast cancer. *Firmicutes* and *Fusobacteria* were overexpressed and the beneficial bacteria were reduced in patients who suffer from the colorectal cancer. *Proteobacteria* are the dominant bacteria in pancreatic cancer. The presence of *Fnucleatum* in esophageal cancer tissue is associated with a poor prognosis. *E. coli* and *Enterococcus* increased in patients who suffer from the liver cancer (Zhou et al., 2020).

## Immunotherapy

Currently, the known immunotherapies include cytokines, T cells (checkpoint inhibitors, costimulatory receptor agonists), T cell modification, oncolytic viruses, therapies targeting other cell types, and vaccines (Lizee et al., 2013; Baumeister et al., 2016; Johnson and June, 2017; Allahverdiyev et al., 2018), among which, ICIs monotherapy or combination therapy, has been observed with sustained remission and significant survival advantage in a variety of solid tumors, and is approved for first-line or second-line treatment in a variety of tumors. These include melanoma, renal cell carcinoma (RCC), hepatocellular carcinoma, urothelial carcinoma, head and neck carcinoma, non-small cell lung cancer (NSCLC), and gastric cancer (Topalian et al., 2012; Larkins et al., 2017). In recent years, ICIs have become a new milestone in improving the clinical treatment of cancer. For example, for the lung cancer, the immunotherapy has been applied from the second line to the first line; from metastatic lung cancer to locally advanced, to neoadjuvant therapy, and then to adjuvant therapy; and from highly selective monotherapy to combination therapy. For the gastric cancer, since 2018, the recommendation level of immunotherapy guidelines has been moving forward from third-line treatment to first-line treatment.

Although immunotherapy has many advantages, we found that it still has certain limitations, which include the low overall treatment response rate, acquired drug resistance, and immunotherapy related to adverse reactions (Murray, 1990; Russo and Johnson, 2003; Kaper et al., 2004). At present, the methods to predict the effect of immunotherapy are mainly determined by gene sequencing and pathological examination, including the expression of PD-1 / PD-L1, microsatellite status, tumor mutation load. However, these methods do not screen out the population that can benefit from immunotherapy well, and the gut microbiota has shown to be associated with the efficacy of immunotherapy in some preclinical and clinical studies (Sivan et al., 2015; Chaput et al., 2017; Routy et al., 2018), making it a possible new target for predicting immunotherapy sensitivity.

## Gut Microbiota and Immunotherapy

### Gut Microbiota Affects the Efficacy of Immunotherapy

Based on the existing preclinical and clinical studies, we found that the gut microbiota affects the efficacy of immunotherapy. In 2015, Ayelet Sivan et al. found that *Bifidobacteria* can enhance anti-tumor immunity in mice by comparing the growth of melanoma in mice with different commensal microbiota and the differences in spontaneous anti-tumor immunity (Sivan et al., 2015). This research revealed a link between the gut microbiota and the efficacy of ICIs. In order to further confirm the role of gut microbiota in regulating the efficacy of immunotherapy for melanoma, lots of researchers have carried out the clinical trials. Matson et al. analyzed fecal samples from 42 patients with metastatic melanoma before immunotherapy, of which 16 patients responded and 26 patients failed to do so. According to the studies, it has found that the *Bifidobacterium longum, Collinsella aerofaciens,* and *Enterococcus faecium* have higher relative abundance in patients responding to PD-1 inhibitors. It was further found that reconstructing sterile mice with feces from responding patients improved tumor control, enhanced T cell response, and improved the efficacy of PD-1 inhibitors therapy (Matson et al., 2018). Chaput et al. compared the stools of 26 patients with melanoma treated with Ipilimumab and found that patients with a baseline predominance of *Faecalibacterium* and other *Firmicutes* had longer Progression free survival (PFS) than those with a predominance of *Bacteroidetes* at baseline (Chaput et al., 2017). The study published by Frankel et al. included 39 patients with metastatic melanoma who received different types of immunotherapy, and found that ICIs responders to all types of treatment were enriched with *Bacteroides caccae*. For the responders who are treated with a combination of the anti-CTLA4 and anti-PD-1 immune checkpoint blockade, the intestinal microbiome of *Faecalibacterium Prausnitzii, Bacteroides thetaiotamicron,* and *Holdemania filiformis* were enriched (Frankel et al., 2017). In order to further explore the relationship, the researchers investigated the same in other cancers. Jing et al. compared the stools of 37 NSCLC patients treated with nivolumab and found that responders had high abundances of *B. longum, Alisberia* and *Prevotella* (Jin et al., 2019). Routly et al. found that in the gut microbiota of patients with NSCLC and kidney cancer treated with PD-1 inhibitors, the levels of *Akkermansia* in the stool of responders were significantly higher than those of non-responders (Routy et al., 2018). Gopalakrishnan et al. compared the feces of 112 melanoma patients treated with PD-1 inhibitors and found that responders were rich in *Faecalibacterium* and *Ruminococcus*, and non-responders were rich in *Bacteroides* (Gopalakrishnan et al., 2018). Yi Zheng et al. also revealed the relationship between the specific gut microbiota and immunotherapy efficacy in liver cancer. The study found that in patients with hepatocellular carcinoma receiving PD-1 inhibitor therapy, the gut microbiota of responders was higher in *Akkermansia* and *Ruminococcus* (Zheng et al., 2019). These preclinical and clinical pieces of evidence support the role of gut microbiota in modulating the efficacy of immunotherapy for various cancers.

### The Effect of Antibiotics on Immunotherapy

Lots of studies have shown that many factors can affect the microecological balance of the gut microbiota, including delivery mode, host genetics, age, diet, infection, antibiotics(ATB) and so on (Dominguez-Bello et al., 2016; Zhernakova et al., 2016; Rothschild et al., 2018). Clinically, we pay more attention to the effect of antibiotic use on microbiota. Antibiotics control the infection, while reducing the diversity of bacteria, which can lead to intestinal dysbiosis. Disrupted gut microbiota, in turn, affects the immune system. Routy et al. reviewed 140 patients with advanced NSCLC, 67 patients with RCC, and 42 patients with urothelial carcinoma. Data showed that PFS and overall survival (OS) were significantly reduced in the ATB-treated group when all patients were combined (median PFS: 3.5 vs. 4.1 months; median OS: 11.5 vs. 20.6 months); for patients who suffer from the advanced NSCLC, OS was shorter in the ATB-treated group (median OS:8.3 vs. 15.3 months); for the patients who suffer from the RCC, PFS in the ATB-treated group was also shorter (median PFS:4.3 vs. 7.4 months; Routy et al., 2018). Hakozaki et al. retrospectively analyzed data from 90 patients with NSCLC who received nivolumab and found that 13 patients had received ATB prior to nivolumab. Data showed that patients with NSCLC who received ATB before nivolumab had significantly shorter PFS and OS (median PFS: 1.2 vs. 4.4 months; median OS: 8.8 vs. NR months, *p* = 0.037; Hakozaki et al., 2019). Wilson et al. conducted a meta-analysis of patients with various tumor types who were primarily treated with PD-1 inhibitors or PD-L1 inhibitors. Data showed that patients who did not use ATB before or during immunotherapy had longer OS and PFS (Wilson et al., 2020). Another study included 568 patients, of whom 114 (20.1%) had received ATB treatment prior to ICIs. Data showed that the patients the OS of the antibiotic exposed group was significantly worse than that of the unexposed group. The median survival was shorter among all patients in the exposed group than in the unexposed group (mPFS: 27.4 vs. 43.7 months; Mohiuddin et al., 2021). Contrary to these findings, a study by Kaderbhai et al. showed that antibiotic administration had no effect on PFS in nivolumab-treated NSCLC patients (Kaderbhai et al., 2017). Due to limited clinical data at present, the impact of antibiotic use on immunotherapy needs to be assessed with large sample sizes.

### Mechanism of Interaction Between the Gut Microbiota and Immune System

The gut microbiota may interact with the body through a variety of different mechanisms, affecting the body’s immune system and regulating the effect of immunotherapy. The current known main mechanisms include: (1) Bacterial metabolites enter the circulation and bind to host cells through receptors, thus affecting the host immune system. Mager et al. have now revealed that the mechanism between the mouse gut microbiome and response to ICIs is the presence of a microbiologically produced purine riboside molecule called inosine (Mager et al., 2020). Inosine, an intestinal metabolite produced by *Bifidobacterium* and *Akkermansia muciniphila*, enhanced Th1 differentiation and effector function of A2AR expressing naive T cells, never enhancing anti-CTLA-4 and anti-PD-L1 therapy. Meanwhile, the current study found that bacteria associated with improved immunotherapy efficacy produce short-chain fatty acids (SCFAs). Short-chain fatty acids are the major terminal metabolites produced by gut microbes (Macfarlane and Macfarlane, 2003). Higher fecal and plasma SCFA concentrations of immunotherapy responders were associated with longer PFS (Nomura et al., 2020). The short-chain fatty acids have immunomodulatory functions. For example, butyric acid has been shown to induce differentiation of Foxp3 + CD4 + Treg (Arpaia et al., 2013; Furusawa et al., 2013; Smith et al., 2013). Butyric acid and other SCFAs increase the expression of IFNγ and granzyme B in CD8 + cytotoxic T lymphocytes and interleukin-17 secreting CD8 + T cells (Luu et al., 2018). (2) Pathogen-associated molecular patterns (PAMPs) such as lipopolysaccharides are released by the gut microbiota and regulate immune function by activating pattern recognition receptors such as toll-like receptors (TLRs; Janeway, 1989; Lathrop et al., 2011; Stary et al., 2015). Commensal bacteria can prime dendritic cells (DCs), which in turn can signal Toll-like receptors (TLRs) to train the immune system regarding differential recognition of pathogenic versus nonpathogenic microbes (Michelsen et al., 2001; van Kooyk and Geijtenbeek, 2003; Minarrieta et al., 2017). The gut microbiota promotes the maturation of local gut-associated lymphoid tissue (GALT) and induces B cell differentiation, maturation, and activation (Fagarasan et al., 2002; Ouwehand et al., 2002; Wei et al., 2008; Lundell et al., 2012). Furthermore, the gut microbiota regulates the systemic immune response and alters immune cell activity *via* soluble immunoregulatory factors and circulating cytokines. By destroying the integrity of the intestinal wall, activating immune pathways, recruiting immune cells, and initiating anti-cancer immune responses, ICIs can promote the transfer of microorganisms to distant tumor tissues, thereby enhancing the immune activation effect of intestinal microbiota on the systemic system (Almonte et al., 2021).

By reviewing such preclinical and clinical trials, we found a relationship between the microbiome and the immune system. However, the specific mechanism of the way that the intestinal microbiota influences the immune system, is still not clear and, needs further discussion in the future.

## Regulating the Gut Microbiota

Current preclinical and clinical evidence suggests that the presence and composition of gut microbiota plays a role in immunotherapy. On this basis, we propose a strategy to regulate immunotherapy by manipulating gut microbiota. The known methods mainly include fecal microbiota transplantation, dietary regulation, prebiotics, etc.

### Fecal Microbiota Transplantation

Fecal microbiota transplantation (FMT), also known as fecal transplantation, is the process of placing stool from a healthy donor into the gut of another patient (Smits et al., 2013; Liang et al., 2014). Previous research has established that FMT is a clinically effective method of restoring gut microbiota for the treatment of recurrent *C. difficile* infections (van Nood et al., 2013; Cammarota et al., 2015; Costello et al., 2015). There is also evidence that FMT can help with the treatment of inflammatory bowel disease (Moayyedi et al., 2015; Paramsothy et al., 2017; Costello et al., 2019). Furthermore, a large number of clinical studies are being conducted to investigate the role of FMT in tumor immunotherapy. Matson et al. discovered that using feces from immunotherapy responders to rebuild the intestinal microbiota of germ-free mice could control tumor growth and improve immune efficacy (Ouwehand et al., 2002). Routy et al. reached a similar conclusion, claiming that FMT from ICIs-responsive cancer patients into sterile or antibiotic-treated mice improved immunotherapy efficacy (Routy et al., 2018). Two recent Phase 1 clinical studies suggest that FMT may be able to overcome immunotherapy resistance (Baruch et al., 2021; Davar et al., 2021). Erez N et al. enrolled 10 patients with metastatic melanoma who were unresponsive to anti-PD-1 therapy in a phase I clinical trial. Anti-PD-1 immunotherapy and FMT were given to the patients by donors who had a complete response to metastatic melanoma immunotherapy. Clinical responses were observed in three patients, with two showing partial responses and one showing a complete response (Baruch et al., 2021). Davar et al. obtained similar results (Davar et al., 2021). Based on the findings above, we believe that FMT combined with ICIs can improve patient response to immunotherapy. However, the optimal dose, route of administration, and screening criteria of donor stool are still unknown in terms of the efficacy and safety of FMT treatment, and more large-sample, high-quality studies are needed to investigate.

### Diet

Diet is an important factor that affects the gut microbiota. The researchers found differences in the structure of the gut microbiota with different eating habits (David et al., 2014; Gentile and Weir, 2018). The intestinal microbiota of vegetarians is dominated by *Clostridium coccoides* and *Clostridium ramosum* (Hayashi et al., 2002). The dominant bacteria in the intestinal tract of people with long-term high levels of meat consumption were *Faecalibacterium prausnitzii* (Mueller et al., 2006). A high-fat diet will reduce the number of *Bacteroidetes* and *Bifidobacterium*, and increase the number of *Firmicutes* and *Proteobacterium* (Zhang et al., 2012). A high-protein diet can make *Bacteroidetes* and *Bacillus bicinophilus* proliferate in the intestines, which may lead to reduced human immunity and increased disease risk (including metabolic diseases; Forouhi et al., 2018). Therefore, we can improve the structure of intestinal microbiota and create a more favorable microecology by adjusting our diet.

### Functional Foods

Functional foods such as prebiotics are conducive to the functional stability of the small intestine and colon (Aguilar-Toala et al., 2020), and the metabolism of these substances by intestinal microbiota can improve the gastrointestinal function and barrier homeostasis, enhance the mineral absorption capacity of the human body, regulate energy metabolism and reduce the risk of intestinal pathogenic bacteria infection (Sanders et al., 2019).

Probiotics are a class of active microorganisms that are beneficial to the host by colonizing the human body and changing the composition of a certain part of the host’s microbiota. It can protect the intestinal mucosal barrier, maintain the balance of intestinal microbiota structure, and improve the immunity of the body. At present, the most commonly used probiotics mainly include *Bifidobacterium, lactobacillus*, and yeast. Probiotics can regulate the acid–base balance of the intestinal environment by producing SCFAs. In addition, during the metabolic process, probiotics can also regulate the activity of macrophages, cytokines, and immunoglobulin levels to activate the immune response (La Fata et al., 2018). Some scholars proposed that immunotherapeutic probiotics could be developed to improve the immune efficacy (Dai et al., 2020), though their practical application is still hindered in lots of aspects.

## Conclusion

At present, a number of preclinical trials and clinical trials have demonstrated that intestinal microbiota influences tumor development and host immune response. It is still unknown which specific bacterial is most closely related to the occurrence and development of tumors, which bacterial is most conducive to promoting immune efficacy, and how to rationally use antibiotics is the most appropriate for patients, which need to be further explored in clinical trials. Intestinal microbiota is susceptible to a variety of factors, and the comparability between the results of studies is limited due to the differences in sequencing analysis techniques, regional differences in subjects, dietary and lifestyle habits, use of other therapeutic drugs, gender and age, etc. Therefore, these factors can be taken into account in subsequent clinical studies to make the relationship between microbiota and immunotherapy clearer. At present, the effective rate of immunotherapy is not high, so how to screen out the people who can benefit from immunotherapy has become the clinical focus. Detection of biomarkers is the cornerstone of precision immunotherapy. In recent years, tumor genomic markers, tumor immune microenvironment markers, host germline genetic markers, systemic blood circulation markers and other markers have been paid attention to and explored, but no biomarkers with high sensitivity and specificity have been found. In future studies, the relationship between intestinal microbiota and immunotherapy can be further studied to make it a new prediction target. At the same time, a variety of biomarkers can be jointly detected to improve the accuracy of screening population and make the immunotherapy precise and individualized.

## Author Contributions

XX performed the literature search, wrote the manuscript, and guaranteed its integrity. JY conceived the framework of the manuscript and revised the entire manuscript. All authors contributed to the article and approved the submitted version.

## Funding

This project was funded by Program of Zhejiang Provincial TCM Sci-tech Plan (no. 2021ZZ005) and Medical Health Plan of Zhejiang Province (no. 2022KY666).

## Conflict of Interest

The authors declare that the research was conducted in the absence of any commercial or financial relationships that could be construed as a potential conflict of interest.

## Publisher’s Note

All claims expressed in this article are solely those of the authors and do not necessarily represent those of their affiliated organizations, or those of the publisher, the editors and the reviewers. Any product that may be evaluated in this article, or claim that may be made by its manufacturer, is not guaranteed or endorsed by the publisher.
